# 5-Hydroxycyclopenicillone Inhibits β-Amyloid Oligomerization and Produces Anti-β-Amyloid Neuroprotective Effects In Vitro

**DOI:** 10.3390/molecules22101651

**Published:** 2017-10-01

**Authors:** Jiaying Zhao, Fufeng Liu, Chunhui Huang, Jieyi Shentu, Minjun Wang, Chenkai Sun, Liping Chen, Sicheng Yan, Fang Fang, Yuanyuan Wang, Shujun Xu, C. Benjamin Naman, Qinwen Wang, Shan He, Wei Cui

**Affiliations:** 1 Li Dak Sum Yip Yio Chin Kenneth Li Marine Biopharmaceutical Research Center, Ningbo University, Ningbo 315211, China; 156001372@nbu.edu.cn (J.Z.); 156001359@nbu.edu.cn (C.H.); 156001362@nbu.edu.cn (J.S.); 156001366@nbu.edu.cn (M.W.); 156001364@nbu.edu.cn (C.S.); 15888107865@163.com (F.F.); bnaman@ucsd.edu (C.B.N.); 2 Ningbo Key Laboratory of Behavioral Neuroscience, Zhejiang Provincial Key Laboratory of Pathophysiology, School of Medicine, Ningbo University, Ningbo 315211, China; ChenLiliy@outlook.com (L.C.); YanSicheng9@163.com (S.Y.); 1511075182@nbu.edu.cn (Y.W.); Xushujun@nbu.edu.cn (S.X.); Wangqinwen@nbu.edu.cn (Q.W.); 3 Key Laboratory of Industrial Fermentation Microbiology of Education, College of Biotechnology, Tianjin University of Science & Technology, Tianjin 300457, China; fufengliu@tust.edu.cn; 4 Center for Marine Biotechnology and Biomedicine, Scripps Institution of Oceanography, University of California, San Diego, La Jolla, CA 92093, USA

**Keywords:** 5-hydroxycyclopenicillone, β-amyloid, Alzheimer’s disease, sponge-associated fungus, oligomer

## Abstract

The oligomer of β-amyloid (Aβ) is considered the main neurotoxin in Alzheimer’s disease (AD). Therefore, the inhibition of the formation of Aβ oligomer could be a target for AD therapy. In this study, with the help of the dot blotting assay and transmission electronic microscopy, it was have discovered that 5-hydroxycyclopenicillone, a cyclopentenone recently isolated from a sponge-associated fungus, effectively reduced the formation of Aβ oligomer from Aβ peptide in vitro. Molecular dynamics simulations suggested hydrophobic interactions between 5-hydroxycyclopenicillone and Aβ peptide, which might prevent the conformational transition and oligomerization of Aβ peptide. Moreover, Aβ oligomer pre-incubated with 5-hydroxycyclopenicillone was less toxic when added to neuronal SH-SY5Y cells compared to the normal Aβ oligomer. Although 5-hydroxycyclopenicillone is not bioavailable in the brain in its current form, further modification or encapsulation of this chemical might improve the penetration of 5-hydroxycyclopenicillone into the brain. Based on the current findings and the anti-oxidative stress properties of 5-hydroxycyclopenicillone, it is suggested that 5-hydroxycyclopenicillone may have potential therapeutic efficacy in treating AD.

## 1. Introduction

Alzheimer’s disease (AD) is characterized by the reduction of cognitive functions and the loss of neurons in the brain, and is the most prevalent neurodegenerative disorder worldwide [1]. Unfortunately, there is no effective treatment available for this disease. Although the pathogenesis of AD is still unclear, many studies have suggested that the β-amyloid (Aβ) oligomer, formed by the self-aggregation of the Aβ peptide, is the main neurotoxin of AD [2,3]. The Aβ oligomer can induce neuronal death, leading to the cognitive impairments in AD patients. Therefore, it is generally accepted that the Aβ oligomer should be a primary target for AD therapy [4].

Previous studies have shown that many compounds can inhibit the formation of the Aβ oligomer [5,6,7]. For example, curcumin, a natural diarylheptanoid derived from *Curcuma longa*, can block the Aβ oligomer formation and increase cognition in animals with AD [5]. Brazilin, a pigment obtained from brazilwood (*Caesalpinia* sp.), inhibits Aβ assembly and prevents neuronal death in vitro [6]. Ocrein, a natural dye extracted from lichen, stops the growth of the Aβ oligomer and prevents the decrease of long-term potentiation in hippocampal slices [7]. These results strongly suggest that natural compounds with the ability to inhibit the formation of the Aβ oligomer might prevent Aβ oligomer-induced neurotoxicity, and could be developed or used for AD therapy.

Marine organisms are important sources of structurally diverse compounds with a wide variety of biological activities [8]. Interestingly, many natural compounds derived from ocean organisms can also modulate Aβ oligomer formation. For instance, an aqueous extract from winged kelp (*Alaria esculenta*) inhibits the formation of amyloid aggregation [9]. Fucoxanthin is a marine carotenoid derived from edible blown algae, which inhibits Aβ oligomer formation and was shown to reverse cognitive impairments in animals with AD [10,11]. These and other results highlight the possibility that marine compounds might be used in AD treatment.

A newly discovered cyclopentenone from a sponge-associated fungal strain *Trichoderma* sp. HPQJ-34, 5-hydroxycyclopenicillone, possesses anti-oxidative properties and produces neuroprotective effects in vitro against H_2_O_2_-induced neuronal death [12]. In this study, the effects of 5-hydroxycyclopenicillone on the formation of the Aβ oligomer were evaluated, and the interaction between 5-hydroxycyclopenicillone and Aβ peptide were explored by molecular dynamics (MD) analysis. The neurotoxicity of the 5-hydroxycyclopenicillone-modifed Aβ oligomer in SH-SY5Y cells was also investigated.

## 2. Results

### 2.1. 5-Hydroxycyclopenicillone Inhibits Aβ_1-42_ Oligomer Formation

**Figure 1:**
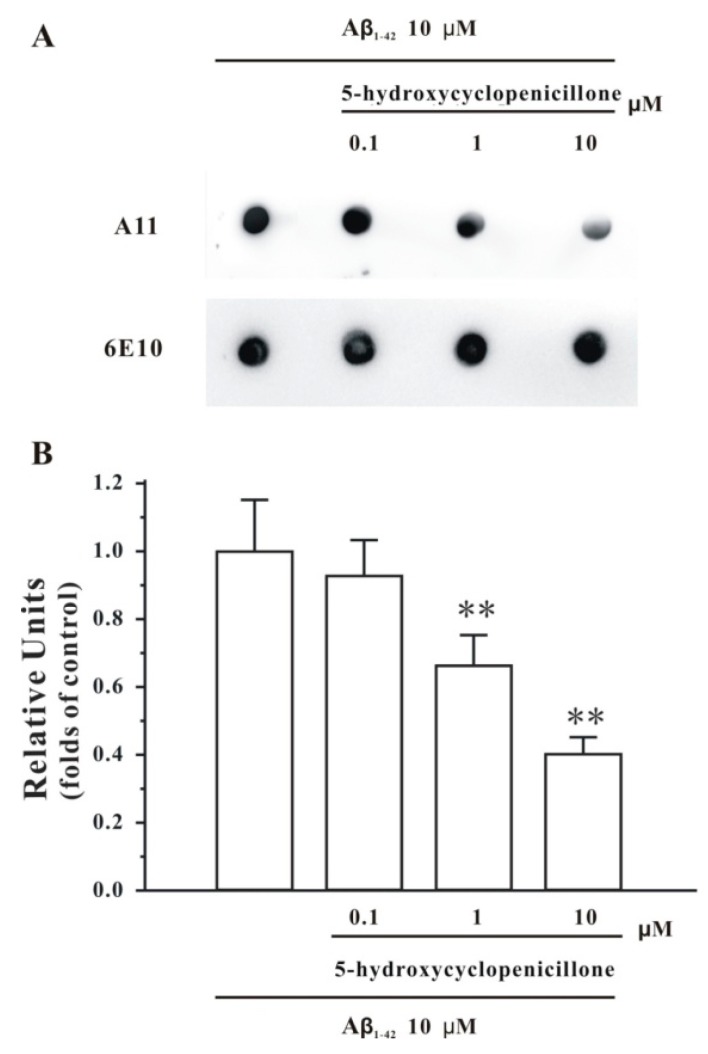
5-Hydroxycyclopenicillone reduces Aβ_1-42_ oligomer formation in a dose-dependent manner. The Aβ_1-42_ peptide was co-incubated with 5-hydroxycyclopenicillone at the indicated doses for 2 days. (**A**) Solution was centrifuged, and the supernatants were examined via dot blotting analysis with A11, an anti-oligomer antibody, and 6E10, an anti-Aβ antibody, respectively. (**B**) The bands from three independent experiments were quantified via densitometry and represented graphically. Data were the mean ± SEM; ** *p* < 0.01 versus the control group (ANOVA and Tukey’s test).

To evaluate whether 5-hydroxycyclopenicillone affects Aβ oligomerization, the in vitro dot blotting assay was used with the Aβ_1-42_ peptide. In a control test, the Aβ_1-42_ peptide formed the Aβ_1-42_ oligomer after two days of incubation under stirring. However, co-incubation of 5-hydroxycyclopenicillone at 1 and 10 μM significantly (*p* < 0.01) reduced the amounts of the Aβ_1-42_ oligomer formed compared to the control condition (Figure 1).

**Figure 2:**
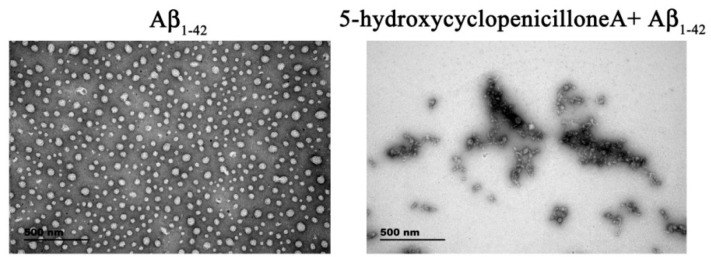
5-Hydroxycyclopenicillone inhibits Aβ_1-42_ oligomer formation. Aβ_1-42_ peptide (10 μM) was co-incubated with 10 μM 5-hydroxycyclopenicillone for 2 days. Solution was centrifuged, and the supernatants were examined via TEM.

Furthermore, transmission electron microscopy (TEM) was used to evaluate the morphology of the 5-hydroxycyclopenicillone-modified Aβ_1-42_ oligomer. The Aβ_1-42_ peptide (10 μM) was co-incubated with or without 10 μM 5-hydroxycyclopenicillone for 2 days. The globular Aβ_1-42_ oligomer with a diameter of about 10–80 nm was the main constituent in the control sample. These results were consistent with many previous reports [13,14,15,16]. Interestingly, small amounts of chain-like aggregates were found in the sample after co-incubation of the Aβ_1-42_ peptide with 5-hydroxycyclopenicillone, providing more evidence that 5-hydroxycyclopenicillone inhibits Aβ_1-42_ peptide oligomerization (Figure 2).

### 2.2. 5-Hydroxycyclopenicillone Likely Binds to Aβ_1-42_ Peptides via Hydrophobic Interactions

**Figure 3:**
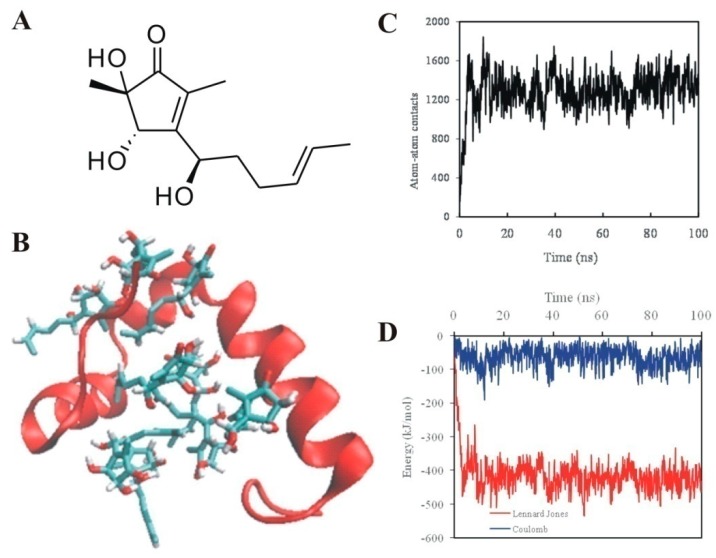
5-Hydroxycyclopenicillone binds to the Aβ_1-42_ peptide via hydrophobic interactions in all-atom molecular dynamics simulations. (**A**) Chemical structure of 5-hydroxycyclopenicillone. (**B**) Typical binding conformations of 5-hydroxycyclopenicillone interacted with the Aβ_1-42_ peptide. The backbone of the Aβ_1-42_ peptide is shown in red. 5-Hydroxycyclopenicillone molecules are shown with a stick model. Red represents oxygen, white represents hydrogen, and green represents carbon. (**C**) Time dependence of atom contacts between the Aβ_1-42_ peptide and 5-hydroxycyclopenicillone molecules (within 0.5 nm). (**D**) Analysis of Lennard-Jones and coulomb energies between the Aβ_1-42_ peptide and 5-hydroxycyclopenicillone. The results were all averaged for three trajectories.

To probe the interaction between 5-hydroxycyclopenicillone and Aβ aggregation, all-atom molecular dynamics simulations were performed. The molecular structure of 5-hydroxycyclopenicillone is shown in Figure 3A. It was suggested that several molecules of 5-hydroxycyclopenicillone can interact with the Aβ_1-42_ peptide, forming a stable conformation that may prevent the conformational transition and oligomerization of the Aβ_1-42_ peptide (Figure 3B). The number of atomic contacts observed in the simulation between 5-hydroxycyclopenicillone and the Aβ_1-42_ peptide increased from the initial 180 to around 1600 within the first 5 ns. Furthermore, the average number of atomic contacts between 5-hydroxycyclopenicillone and the Aβ_1-42_ peptide was over 1200 during the course of the simulation. The intermolecular Lennard-Jones and electrostatic energies between 5-hydroxycyclopenicillone and the Aβ_1-42_ peptide was calculated. The Lennard-Jones energy mainly contributed to the interactions between 5-hydroxycyclopenicillone and the Aβ_1-42_ peptide, indicating that 5-hydroxycyclopenicillone most likely bound to Aβ_1-42_ peptide via hydrophobic interactions.

### 2.3. 5-Hydroxycyclopenicillone Decreased the Neurotoxicity of the Aβ_1-42_ Oligomer in SH-SY5Y Cells

**Figure 4:**
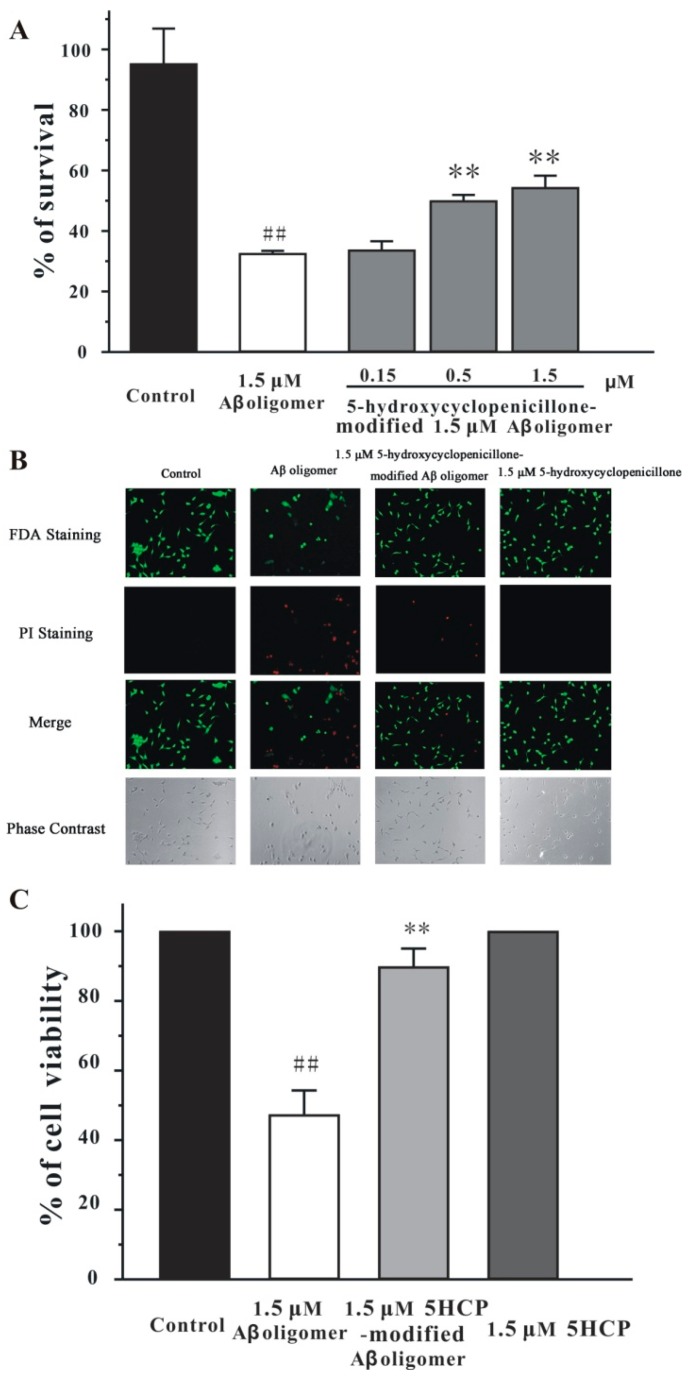
5-Hydroxycyclopenicillone-modified Aβ_1-42_ oligomer is less toxic than normal oligomer to SH-SY5Y cells. (**A**) Aβ_1-42_ peptide (1.5 μM) was co-incubated with 5-hydroxycyclopenicillone at the indicated doses for 2 days. The solution was centrifuged. The supernatants were added to SH-SY5Y cells. After 24 h, the MTT assay was used to analyze cell viability. (**B**) SH-SY5Y cells were treated with the 1.5 μM 5-hydroxycyclopenicillone-modified 1.5 μM Aβ1-42 oligomer or the 1.5 μM Aβ_1-42_ oligomer. After 24 h, cells were examined via FDA/PI double staining. (**C**) The quantification of (**B**). 5HCP: 5-hydroxycyclopenicillone. Data, expressed as percentage of control, were the mean ± SEM of three separate experiments; ## *p* < 0.01 vs. the control group, ** *p* < 0.01 vs. Aβ1-42 oligomer group (ANOVA and Tukey’s test).

The Aβ_1-42_ oligomer is known to induce neuronal death in SH-SY5Y cells [17]. To further explore whether 5-hydroxycyclopenicillone could reduce the neurotoxicity of the Aβ_1-42_ oligomer, it was tested using the 3-(4,5-dimethylthiazol-2-yl)-2.5-diphenyltetrazolium bromide (MTT) assay. Treatment of 1.5 μM Aβ_1-42_ oligomer for 24 h decreased the cell viability to around 40%, compared to the control group. Three concentrations of 5-hydroxycyclopenicillone (0.15–1.5 μM) were co-incubated with 1.5 μM Aβ_1-42_ peptide for 2 days to form the 5-hydroxycyclopenicillone-modified Aβ_1-42_ oligomer. Cell viability in SH-SY5Y cells treated with the 5-hydroxycyclopenicillone (0.5–1.5 μM)-modified Aβ_1-42_ oligomer was substantially higher than that in cells treated with the normal Aβ_1-42_ oligomer (Figure 4A). These results suggested that 5-hydroxycyclopenicillone reduced the formation of oligomer, leading to decreased neurotoxicity. Fluorescein diacetate (FDA)/propidium iodide (PI) double staining was used to label both live cells and dead cells. The number of FDA-labeled live cells was greater in cells treated by the 1.5 μM 5-hydroxycyclopenicillone-modified Aβ_1-42_ oligomer than those treated by the normal Aβ_1-42_ oligomer, and the number of PI-labeled dead cells was smaller (Figure 4B).

### 2.4. 5-Hydroxycyclopenicillone Prevents Synaptic Toxicity of Aβ_1-42_ Oligomer in Primary Hippocampal Neurons

**Figure 5:**
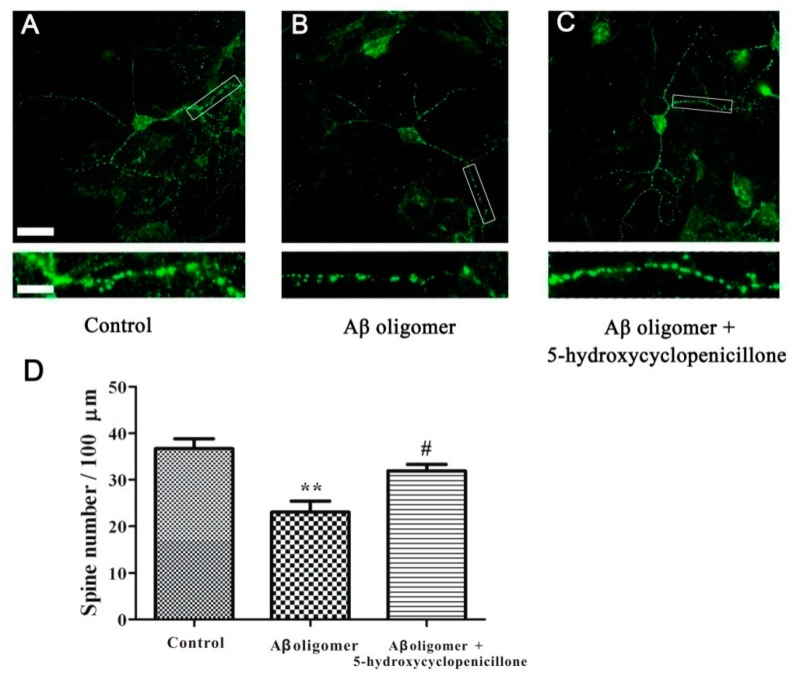
5-hydroxycyclopenicillone significantly prevents Aβ oligomer-induced reduction of spine number in primary hippocampal neurons. At 15 day in vitro (DIV 15), primary hippocampal neurons were treated with 1.5 μM 5-hydroxycyclopenicillone. Two hours later, neurons were treated with 0.5 μM Aβ oligomer. Neurons were fixed and stained with anti-PSD95 antibody at 24 hours after the treatment of Aβ oligomer. The representative spine morphology in control, Aβ oligomer, and 5-hydroxycyclopenicillone + Aβ oligomer groups were shown in (**A**), (**B**) and (**C**), respectively. The quantitative comparison of spine numbers in various groups was demonstrated in (**D**). Data, expressed as the percentage of control, were the mean ± SEM of three separate experiments; ** *p* < 0.01 vs. the control group, # *p* < 0.05 vs. Aβ oligomer group (ANOVA and Tukey’s test).

It was previously demonstrated that Aβ oligomer produces neurotoxicity in primary hippocampal neurons [18,19]. Therefore, the neuroprotective effects of 5-hydroxycyclopenicillone in this model have been here evaluated. It was found that 0.5 μM Aβ oligomer significantly reduced the number of spines in the mature hippocampal neurons, which is consistence with previous reports [19,20]. However, the addition of 1.5 μM 5-hydroxycyclopenicillone significantly prevented the Aβ oligomer-induced reduction of hippocampal neuronal spine numbers, suggesting that 5-hydroxycyclopenicillone could produce neuroprotective effects in primary neurons (Figure 5).

## 3. Discussion

Sponges are marine organisms that are prolific producers of bioactive secondary metabolites, either natively or through their associated microbiota [21]. However, the short supply of sponges and the typically low purification yield of natural products from sponges has prevented the further development of many sponge-derived compounds [22,23]. Interestingly, it has been shown that many sponge-derived bioactive compounds are produced by sponge-associated bacteria or fungi [24,25]. A recently described fungal strain, *Trichoderma* sp. HPQJ-34, was isolated from the marine sponge *Hymeniacidon perleve* [12]. This fungus, in culture, produces 5-hydroxycyclopenicillone, a new cyclopentenone that has in vitro anti-oxidative properties and prevents H_2_O_2_-induced neurotoxicity [12].

The Aβ oligomer is considered the main neurotoxin to induce AD [26]. Moreover, many anti-AD drug candidates target the formation of the Aβ oligomer from the Aβ peptide [27]. Therefore, the potential for 5-hydroxycyclopenicillone to inhibit the formation of the Aβ oligomer was evaluated. 5-Hydroxycyclopenicillone significantly decreased the amount of the Aβ oligomer at 1–10 μM based on dot blotting analysis, but not the Aβ peptide. Moreover, as evidenced by the TEM assay, the quantity and morphology of 5-hydroxycyclopenicillone-modifed Aβ oligomer is significantly different from that of the normal Aβ oligomer. These results show that 5-hydroxycyclopenicillone manipulated the formation of the Aβ oligomer from the Aβ peptide. The inhibitory potency of 5-hydroxycyclopenicillone on Aβ oligomer formation is similar to other marine compounds with this bioactivity, such as brown algae-derived fucoxanthin [10].

It is worthwhile to investigate how 5-hydroxycyclopenicillone interacts with the Aβ peptide to further prevent the formation of the Aβ oligomer. Results from molecular dynamics analysis suggested that 5-hydroxycyclopenicillone may directly interact with Aβ_1-42_ and Aβ_1-40_ peptides via hydrophobic interactions. The computed interaction between 5-hydroxycyclopenicillone and Aβ peptide further inhibited the conformational transition and oligomerization of the Aβ peptide. This in silico result may help to explain the observed in vitro Aβ peptide oligomerization inhibition of 5-hydroxycyclopenicillone.

Furthermore, it is important to evaluate the neurotoxicity of the 5-hydroxycyclopenicillone-modifed Aβ oligomer. SH-SY5Y cells are derived from human neuroblastoma and are sensitive to neurotoxins such as H_2_O_2_, MPP^+^, and the Aβ oligomer [28]. Therefore, SH-SY5Y cells are widely used to screen neuroprotective drug candidates and to study the molecular mechanisms underlying neurotoxin-induced neuronal death [29,30]. Neuronal death in 5-hydroxycyclopenicillone-modifed Aβ oligomer-treated SH-SY5Y cells was significantly less than that in normal Aβ oligomer-treated cells. Moreover, 5-hydroxycyclopenicillone prevented synaptic toxicity of Aβ1-42 oligomer in primary hippocampal neurons. These results indicated that 5-hydroxycyclopenicillone could decrease Aβ oligomer neurotoxicity and may eventually be used in the treatment of AD. However, the in vivo efficacy of 5-hydroxycyclopenicillone in animals with AD remains to be determined. Preliminary results from this investigation showed that i.p. injection of 5-hydroxycyclopenicillone at 10 mg/kg could not result in the detectable 5-hydroxycyclopenicillone in the brain of mice, indicating that 5-hydroxycyclopenicillone might not readily pass the blood–brain barrier (data not shown). However, it is still possible that 5-hydroxycyclopenicillone might metabolize into active metabolites, which could cross the brain–blood barrier and act on targets of AD in the brain.

Although many sponge-derived natural products have been reported to produce neuroprotective effects, most studies have focused on the ability of these compounds to modulate neurotransmitters, reduce oxidative stress, and enhance neurite growth [23]. Comparatively few studies have investigated the anti-Aβ oligomer properties of sponge-related compounds. In this study, it was found that 5-hydroxycyclopenicillone, a new cyclopentenone derived from sponge-associated fungus, effectively inhibited the formation of the Aβ oligomer from the Aβ peptide. When the Aβ peptide did oligomerize in the presence of 5-hydroxycyclopenicillone, the resulting oligomer was significantly less toxic to SH-SY5Y cells than the normal Aβ oligomer. Molecular dynamics simulations suggested that several hydrophobic interactions between 5-hydroxycyclopenicillone and the Aβ peptide may inhibit the conformational transition and oligomerization of the Aβ peptide. These results together help to explain the recently reported in vitro neuroprotective and Aβ fibrillization inhibiting effects of 5-hydroxycyclopenicillone [12].

## 4. Materials and Methods

### 4.1. Preparation of 5-Hydroxycyclopenicillone

5-Hydroxycyclopenicillone was isolated from the fungal strain *Trichoderma* sp. HPQJ-34 as previous described [12]. Briefly, a culture medium was filtered under reduced pressure to afford the filtrate and mycelia. The filtrate was further extracted by EtOAc to afford the crude extract, which was subjected to silica gel column chromatography eluted with a petroleum ether-EtOAc step gradient to yield several fractions that were combined based on TLC analysis. The 5-hydroxycyclopenicillone-rich fraction was separated by Sephadex LH-20 gel filtration chromatography, eluting with CH_3_OH, to yield several sub-fractions. Semi-preparative HPLC was performed on a Waters HPLC instrument equipped with a Waters RID-10A detector and a C18 column (250 mm × 20 mm ID, 5 µm; YMC Co. Ltd. Tokyo, Japan). The subfraction containing 5-hydroxycyclopenicillone was further purified via reverse-phase semi-preparative HPLC with CH_3_OH/H_2_O (70:30, *v*/*v*) at 2 mL/min to yield 5-hydroxycyclopenicillone. The purity of 5-hydroxycyclopenicillone was greater than 95% as determined by HPLC and ^1^H-NMR analysis.

### 4.2. Preparation of the Aβ_1-42_ Oligomer

Synthetic Aβ_1-42_ peptide was obtained from GL Biochem (Shanghai, China). Soluble Aβ_1-42_ oligomers were prepared as previously described [19]. Briefly, Aβ_1-42_ lyophilized powder was dissolved in hexafluoroisopropanol (HFIP, Sigma, St Louis, MO, USA) to form the Aβ_1-42_ peptide. The Aβ_1-42_ peptide was further spin-vacuumed in 10% HFIP/MilliQ water solution. Subsequently, HFIP was evaporated to obtain the Aβ_1-42_ solution. Next, 10 μL of Aβ_1-42_ solution was added to 40 μL of drug solution. The mixture was incubated at 25 °C for 2 days while stirring and then centrifuged at 14,000× *g* for 15 min at 4 °C. The supernatant consisting mainly of soluble Aβ_1-42_ oligomer was collected.

### 4.3. Dot Blotting Analysis

Dot blotting analysis was performed as previously described [19]. Briefly, the nitrocellulose membrane was divided into equal grids. Subsequently, a 2 μL sample was spotted onto the membrane and then air-dried. The membrane was blocked in a TBST (50 mM Tris, 150 mM NaCl, and 0.1% Tween-20) solution containing 10% milk overnight and then incubated with anti-oligomer antibody A11 (Thermo Fisher Scientific, Waltham, MA, USA, 1:1000) or anti-Aβ1-17 antibody 6E10 (Sigma, 1:1000) for 1 h with gentle shaking. After three washes with TBST, the membrane was incubated with secondary antibodies for 1 h and developed with an enhanced chemiluminescence plus kit.

### 4.4. TEM Analysis

TEM analysis was performed as previously described [19]. Briefly, TEM samples were prepared by placing 2 μL of the pre-incubated solution onto a carbon-coated grid. The samples were stained with 1% uranylacetate and then placed onto a clean paper to remove excess staining solution. The grids were thoroughly examined using a TEM (JEOL, Tokyo, Japan).

### 4.5. Simulation System

The initial structure of Aβ_1-42_ was from the Protein Data Bank (PDB code: 1Z0Q) [31]. Moreover, the 3D structure of 5-hydroxycyclopenicillone was produced by the program Sybyl 6.92. The GROMOS96 53a6 force field parameters of 5-hydroxycyclopenicillone were defined by Automated Topology Builder and Repository 2.0 webserver (https://atb.uq.edu.au/). The charge groups and atomic charges of 5-hydroxycyclopenicillone were further corrected.

### 4.6. Molecular Dynamics Simulation

Aβ_1-42_ was placed into a 6 nm cubic box in silico. Further, 10 molecules of 5-hydroxycyclopenicillone were randomly located around the peptide. Water was also added into the box, and 2 water molecules were replaced by 2 Na^+^ ions to neutralize the negative charge of the system. The simple point charge (SPC) model was used. An energy minimization of 1000 steps was performed to relax the system. The relaxed system was successively equilibrated for 1 ns under an isochoric-isothermal ensemble using the Berendsen weak-coupling method. Finally, three molecular dynamics simulations of 100 ns under different initial conditions were carried out by assigning different initial velocities on each atom of the system. All of the molecular dynamics were performed at physiological temperature and a pressure of 1 bar.

All-atomic molecular dynamics simulations were performed using the GROMACS 5.1.1 package together with the GROMOS96 53A6 force field. Newton’s classical equations of motion were integrated using the Verlet Leapfrog algorithm with a 2 fs time step. All short-range non-bonded interactions were cut off at 1.4 nm, with dispersion correction applied to energy and pressure to determine the truncation of van der Waals interactions. Long-range electrostatic interactions were calculated with the smooth particle mesh Ewald method using cubic-spline interpolation and a Fourier grid spacing of approximately 0.12 nm. The neighbor list was updated every 10 simulation steps. All bond lengths were constrained with the LINCS algorithm with a relative geometric tolerance of 10-4. Initial velocities were assigned according to a Maxwell distribution. For all simulations, the atomic coordinates were saved every 50 ps for the following analysis.

### 4.7. Molecular Dynamics Simulation Analysis

The auxiliary programs provided by GROMACS 5.1.1 package were used to analyze the simulation trajectories. The program gmx energy was used to calculate the Lenard Jones and coulomb potential energies between 5-hydroxycyclopenicillone and Aβ_1-42_. The number of contacts between 5-hydroxycyclopenicillone and residues within 0.5 nm was calculated by the program gmx mindist. The snapshots were made by Visual Molecular Dynamics (VMD) software version 1.9.2.

### 4.8. Culture of SH-SY5Y Cells

SH-SY5Y cells were maintained in high glucose modified Eagle’s medium (DMEM) supplemented with 10% fetal bovine serum (FBS) and penicillin (100 U/mL)/streptomycin (100 μg/mL) at 37 °C with 5% CO_2_. The medium was refreshed every 2 days. Before experiments, SH-SY5Y cells were seeded in DMEM supplemented with 1% FBS for 24 h.

### 4.9. Cell Viability Measurement

Cell viability was measured by MTT assay based on a published protocol [32,33]. Briefly, 10 μL of MTT (5 mg/mL) was added to each well in 96-well plates. Subsequently, the plates were incubated at 37 °C for 4 h, and 100 μL of solvate (0.01 N HCl in 10% SDS) was added. After 16 h, the absorbance of sample was measured at a wavelength of 570 nm using a reference wavelength of 655 nm.

### 4.10. FDA/PI Double Staining

Viable cells were visualized by the fluorescein formed from FDA by esterase activity in viable cells. Non-viable cells were analyzed by PI staining, which only penetrates the membranes of dead cells. Briefly, the cells were examined after incubation with 10 μg/mL FDA and 5 μg/mL PI for 15 min. Images were acquired using UV light microscopy and compared with those taken under phase-contrast microscopy. To quantitatively evaluate cell viability, images of each well were taken from five randomly selected fields, and the number of FDA-positive and PI-positive cells was counted. The percentage of cell viability was analyzed using the equation as follows: % of cell viability = [number of FDA-positive cells/(number of PI-positive cells + number of FDA-positive cells)] × 100%.

### 4.11. Primary Hippocampal Neuronal Cultures

Use and care of animals followed the guidelines of the Ningbo University Animal Research Advisory Committee (Ningbo, Zhejiang, China). Primary neuronal cultures from postnatal 1-day-old ICR mice were prepared as a previous publication [20]. Briefly, the hippocampi were dissected and digested in 0.25% trypsin (Invitrogen, Carlsbad, CA, USA) for 15 min at 37 °C. Dissociated cells were placed on 35 mm culture dishes which were previously coated with poly-D-lysine (100 μg/mL) at density of 7 × 10^5^ cells/cm^2^. Cultures were maintained in a humidified incubator with 5% CO_2_ at 37 °C. The plating medium was Dulbecco’s Modified Eagle Media (Invitrogen) supplemented with 10% FBS, 10% F-12 (Invitrogen). The medium was changed to Neurobasal medium (Invitrogen) supplemented with 2% B27, 1% glutamine after 24 h. At DIV 5, neurons were treated with 5 μM cytosine arabinofuranoside (Invitrogen) to reduce the growth of glials. Half of the medium was replaced twice per week with Neurobasal medium containing 2% B27 and 1% glutamine.

### 4.12. Immunocyto Chemisty

Primary cultured hippocampal neurons were fixed with 4% paraformaldehyde in PBS for 10 min at room temperature, and permeabilized with 0.01% Triton in PBS for 10 min before treatment with 10% BSA for 1 h at room temperature. Cells were then incubated in primary PSD95 antibody (Chemicon, 1:200) in PBS containing 10% BSA overnight at 4 °C. After washing with PBS three times, cells were incubated with secondary antibody for 1 h at room temperature. Imaging of distal neuronal dendrites was performed with a Fluoview 1000 confocal microscope (Olympus, Tokyo, Japan). The background of images was subtracted, and a single threshold was chosen manually to define clusters so that clusters corresponded to puncta at least two-fold greater intensity than the diffuse fluorescence on the dendrite shaft.

### 4.13. Confocal Imaging and Analysis

After drug treatments, the neurons were maintained in a recording chamber with extracellular solution (148 mM NaCl, 3 mM KCl, 3 mM CaCl_2_, 10 mM HEPES and 8 mM glucose, pH 7.4) at room temperature. To measure spine density, images were acquired in 2-D stack. Spine densities were analyzed using Fluoview-1000 software (Olympus Life Science, Tokyo, Japan). All lengths of the primary and secondary dendritic branches were measured by tracing their extension and the spines were counted manually.

### 4.14. Data Analysis and Statistics

Data were expressed as means ± SEM. Statistical significance was determined by one-way ANOVA and Tukey’s test for post-hoc multiple comparison. *p* < 0.05 was considered statistically significant.

## 5. Conclusions

5-hydroxycyclopenicillone is a natural product produced by the fungus *Trichoderma* sp. HPQJ-34, which was isolated from the microbiome of the marine sponge *Hymeniacidon perleve* [12]. This molecule was reported as having “moderate anti-oxidative, anti-Aβ fibrillization properties and neuroprotective effects” in vitro [12]. The results presented here, from thorough in vitro and *ex vivo* testing, provide evidence that the application of 5-hydroxycyclopenicillone produces neuroprotective effects and inhibits the formation of Aβ_1-42_ oligomer from Aβ_1-42_ peptide, and this is one key neurotoxin that is pertinent to AD. Furthermore, *in silico* modeling of the Aβ_1-42_ peptide in the presence of 5-hydroxycyclopenicillone suggested that this interaction can modulate the peptide conformation, and may furthermore prevent the conformational transition that is necessary for oligomerization. When Aβ peptide did oligomerize in the presence of 5-hydroxycyclopenicillone, the resulting modified oligomer was morphologically different and was significantly less toxic to SH-SY5Y cells than the normal Aβ oligomer. A preliminary in vivo test did not result in detectable levels of 5-hydroxycyclopenicillone in murine brain cells, which may indicate a low blood-brain barrier permeability, an innate instability under physiological conditions, a high rate of metabolism, or suboptimal distribution for this molecule. The current finding that 5-hydroxycyclopenicillone inhibits Aβ oligomer formation and decreases Aβ oligomer neurotoxicity provides support for the application of this and other sponge-related compounds to treat neurodegenerative disorders, particularly including AD, and highlights the need for synthetic medicinal chemistry production and optimization, as well as in vivo validation.

## References

[1] Scheltens P., Blennow K., Breteler M.M.B., de Strooper B., Frisoni G.B., Salloway S., van der Flier W.M. (2016). Alzheimer’s disease. Lancet.

[2] Viola K.L., Klein W.L. (2015). Amyloid beta oligomers in Alzheimer’s disease pathogenesis, treatment, and diagnosis. Acta Neuropathol..

[3] Selkoe D.J., Hardy J. (2016). The amyloid hypothesis of Alzheimer’s disease at 25 years. EMBO Mol. Med..

[4] Salahuddin P., Fatima M.T., Abdelhameed A.S., Nusrat S., Khan R.H. (2016). Structure of amyloid oligomers and their mechanisms of toxicities: Targeting amyloid oligomers using novel therapeutic approaches. Eur. J. Med. Chem..

[5] Thapa A., Jett S.D., Chi E.Y. (2016). Curcumin Attenuates Arnyloid-beta Aggregate Toxicity and Modulates Amyloid-beta Aggregation Pathway. ACS Chem. Neurosci..

[6] Du W.J., Guo J.J., Gao M.T., Hu S.Q., Dong X.Y., Han Y.F., Liu F.F., Jiang S.Y., Sun Y. (2015). Brazilin inhibits amyloid beta-protein fibrillogenesis, remodels amyloid fibrils and reduces amyloid cytotoxicity functional. Sci. Rep..

[7] Bieschke J., Herbst M., Wiglenda T., Friedrich R.P., Boeddrich A., Schiele F., Kleckers D., del Amo J.M.L., Gruning B.A., Wang Q.W. (2012). Small-molecule conversion of toxic oligomers to nontoxic beta-sheet-rich amyloid fibrils. Nat. Chem. Biol..

[8] Ruiz-Torres V., Encinar J.A., Herranz-Lopez M., Perez-Sanchez A., Galiano V., Barrajon-Catalan E., Micol V. (2017). An Updated Review on Marine Anticancer Compounds: The Use of Virtual Screening for the Discovery of Small-Molecule Cancer Drugs. Molecules.

[9] Shanmuganathan B., Malar D.S., Sathya S., Devi K.P. (2015). Antiaggregation Potential of Padina gymnospora against the Toxic Alzheimer’s Beta-Amyloid Peptide(25–35) and Cholinesterase Inhibitory Property of Its Bioactive Compounds. PLoS ONE.

[10] Xiang S.Y., Liu F.F., Lin J.J., Chen H.X., Huang C.H., Chen L.P., Zhou Y.Y., Ye L.Y., Zhang K., Jin J.K. (2017). Fucoxanthin Inhibits beta-Amyloid Assembly and Attenuates beta-Amyloid Oligomer-Induced Cognitive Impairments. J. Agric. Food Chem..

[11] Bryukhovetskiy I., Lyakhova I., Mischenko P., Milkina E., Zaitsev S., Khotimchenko Y., Bryukhovetskiy A., Polevshchikov A., Kudryavtsev I., Khotimchenko M. (2017). Alkaloids of fascaplysin are effective conventional chemotherapeutic drugs, inhibiting the proliferation of C6 glioma cells and causing their death in vitro. Oncol. Lett..

[12] Fang F., Zhao J.Y., Ding L.J., Huang C.H., Naman C.B., He S., Wu B., Zhu P., Luo Q.J., Gerwick W.H. (2017). 5-Hydroxycyclopenicillone, a New beta-Amyloid Fibrillization Inhibitor from a Sponge-Derived Fungus Trichoderma sp HPQJ-34. Mar. Drugs.

[13] Li M., Zhao C.Q., Yang X.J., Ren J.S., Xu C., Qu X.G. (2013). In Situ Monitoring Alzheimer’s Disease ss-Amyloid Aggregation and Screening of A ss Inhibitors Using a Perylene Probe. Small.

[14] Lee J., Culyba E.K., Powers E.T., Kelly J.W. (2011). Amyloid-beta forms fibrils by nucleated conformational conversion of oligomers. Nat. Chem. Biol..

[15] Laganowsky A., Liu C., Sawaya M.R., Whitelegge J.P., Park J., Zhao M.L., Pensalfini A., Soriaga A.B., Landau M., Teng P.K. (2012). Atomic View of a Toxic Amyloid Small Oligomer. Science.

[16] Morgado I., Wieligmann K., Bereza M., Ronicke R., Meinhardt K., Annamalai K., Baumann M., Wacker J., Hortschansky P., Malesevic M. (2012). Molecular basis of beta-amyloid oligomer recognition with a conformational antibody fragment. Proc. Natl. Acad. Sci. USA.

[17] Hu S.Q., Wang R., Cui W., Mak S.H., Li G., Hu Y.J., Lee M.Y., Pang Y.P., Han Y.F. (2015). Dimeric bis (heptyl)-Cognitin Blocks Alzheimer’s beta-Amyloid Neurotoxicity Via the Inhibition of A beta Fibrils Formation and Disaggregation of Preformed Fibrils. CNS Neurosci. Ther..

[18] Jiang L.T., Huang M., Xu S.J., Wang Y., An P.Y., Feng C.X., Chen X.W., Wei X.F., Han Y.F., Wang Q.W. (2016). Bis(propyl)-cognitin Prevents beta-amyloid-induced Memory Deficits as Well as Synaptic Formation and Plasticity Impairments via the Activation of PI3-K Pathway. Mol. Neurobiol..

[19] Chang L., Cui W., Yang Y., Xu S.J., Zhou W.H., Fu H.J., Hu S.Q., Mak S.H., Hu J.W., Wang Q. (2015). Protection against beta-amyloid-induced synaptic and memory impairments via altering beta-amyloid assembly by bis(heptyl)-cognitin. Sci. Rep-Uk.

[20] Xu S.J., Liu G.L., Bao X.M., Wu J., Li S.M., Zheng B.X., Anwyl R., Wang Q.W. (2014). Rosiglitazone Prevents Amyloid-beta Oligomer-Induced Impairment of Synapse Formation and Plasticity via Increasing Dendrite and Spine Mitochondrial Number. J. Alzheimers Dis..

[21] Laport M.S., Santos O.C.S., Muricy G. (2009). Marine Sponges: Potential Sources of New Antimicrobial Drugs. Curr. Pharm. Biotechnol..

[22] Santos O.C.S., Soares A.R., Machado F.L.S., Romanos M.T.V., Muricy G., Giambiagi-deMarval M., Laport M.S. (2015). Investigation of biotechnological potential of sponge-associated bacteria collected in Brazilian coast. Lett. Appl. Microbiol..

[23] Indraningrat A.A.G., Smidt H., Sipkema D. (2016). Bioprospecting Sponge-Associated Microbes for Antimicrobial Compounds. Marinedrugs.

[24] Taylor M.W., Radax R., Steger D., Wagner M. (2007). Sponge-associated microorganisms: Evolution, ecology, and biotechnological potential. Microbiol. Mol. Biol. Rev..

[25] Fuerst J.A. (2014). Diversity and biotechnological potential of microorganisms associated with marine sponges. Appl. Microbiol. Biotechnol..

[26] Jarosz-Griffiths H.H., Noble E., Rushworth J.V., Hooper N.M. (2016). Amyloid- Receptors: The Good, the Bad, and the Prion Protein. J. Biol. Chem..

[27] Hielscher-Michael S., Griehl C., Buchholz M., Demuth H.U., Arnold N., Wessjohann L.A. (2016). Natural Products from Microalgae with Potential against Alzheimer’s Disease: Sulfolipids Are Potent Glutaminyl Cyclase Inhibitors. Marinedrugs.

[28] Li X., Song L., Jope R.S. (1996). Cholinergic stimulation of AP-1 and NF kappa B transcription factors is differentially sensitive to oxidative stress in SH-SY5Y neuroblastoma: relationship to phosphoinositide hydrolysis. J. Neurosci..

[29] Nirmaladevi D., Venkataramana M., Chandranayaka S., Ramesha A., Jameel N.M., Srinivas C. (2014). Neuroprotective effects of bikaverin on H_2_O_2_-induced oxidative stress mediated neuronal damage in SH-SY5Y cell line. Cell. Mol. Neurobiol..

[30] Tian X., Guo L.P., Hu X.L., Huang J., Fan Y.H., Ren T.S., Zhao Q.C. (2015). Protective effects of Arctium lappa L. roots against hydrogen peroxide-induced cell injury and potential mechanisms in SH-SY5Y cells. Cell. Mol. Neurobiol..

[31] Tomaselli S., Esposito V., Vangone P., van Nuland N.A., Bonvin A.M., Guerrini R., Tancredi T., Temussi P.A., Picone D. (2006). The alpha-to-beta conformational transition of Alzheimer’s Abeta-(1–42) peptide in aqueous media is reversible: A step by step conformational analysis suggests the location of beta conformation seeding. ChemBioChem.

[32] Cui W., Zhang Z., Li W., Hu S., Mak S., Zhang H., Han R., Yuan S., Li S., Sa F. (2013). The anti-cancer agent SU4312 unexpectedly protects against MPP(+) -induced neurotoxicity via selective and direct inhibition of neuronal NOS. Br. J. Pharmacol..

[33] Cui W., Zhang Z.J., Hu S.Q., Mak S.H., Xu D.P., Choi C.L., Wang Y.Q., Tsim W.K., Lee M.Y., Rong J.H. (2014). Sunitinib produces neuroprotective effect via inhibiting nitric oxide overproduction. CNS Neurosci. Ther..

